# Supplementary material for: Direct imaging of light-element impurities in graphene reveals triple-coordinated oxygen
Source: Nat Commun. 2019 Oct 8;10:4570. doi: 10.1038/s41467-019-12537-3 (PMC6783479; doi:10.1038/s41467-019-12537-3)
Supplement: Supplementary file 1 — Supplementary Information [file 41467_2019_12537_MOESM1_ESM.pdf]

Direct imaging of light-element impurities in graphene reveals triple-coordinated oxygen

Hofer et. al

Supplementary Discussion

DFT calculations

We used DFT calculations to analyse the differences in structural properties when nitrogen and oxygen are incorporated into graphene. The relaxed models are shown in Supplementary Figure 1. The projected interatomic distances and out-of-plane distances are listed in Supplementary Table 1. Significant differences in the projected positions occur for the pair configuration and the 555-777 DV. In the first case, the two oxygen atoms do not form a bond and stick out of the graphene plane in opposite directions. Their projected distance is larger than the interatomic distance in graphene or the distance between two neighboring nitrogen substitutions. Supplementary Figure 2a compares the experimental data with the projected positions of the pair configuration with oxygen (red atoms) and nitrogen (blue atoms). As clearly visible from the magnified overlay of the model and the STEM image, only the relaxed oxygen configuration fits to the experiment. The same analysis can by applied to the 555-777 DV (Supplementary Figure 2b), where the difference is even larger. Also here, the relaxed configuration with the oxygen atom fits to the STEM image. The other configurations are very similar, except for small variations in the out-of-plane direction, which cannot be distinguished in the projected STEM images.

Supplementary Table 2 shows the energy of the relaxed configurations with the heteroatoms incorporated in the lattice for both, nitrogen and oxygen (E_{in}), the sum of the energies ($E_{out} + E_{isolated}$) when the heteroatoms are released from the lattice (after relaxation) and the isolated

Configurations	Oxygen			Nitrogen		
	r_1 [pm]	r_2 [pm]	z [pm]	r_1 [pm]	r_2 [pm]	z [pm]
Pair	123	186	65	141	145	0
Graphitic	149	-	20	141	-	0
5-9 MV	135	-	1	132	-	0
s-MV	136	264	13	135	266	0
5-8-5 DV	136	242	0	132	248	13
555-777 DV	138	206	22	145	145	0
TV	133	138	3	131	134	0

Supplementary Table 1: Projected interatomic distances and out-of-plane distances of different configurations with oxygen and nitrogen as heteroatoms in graphene. Definitions of r_1 and r_2 are in SFig. 1.

atoms (the half of N_2 or O_2). The difference $E_{in} - (E_{out} + E_{isolated})$ is referred as binding energy and the difference between the binding energies is shown in the last column of the table.

Configurations	Oxygen			Nitrogen			
	E_{in}	$E_{out} + E_{iso}$	E_{bind}	E_{in}	$E_{out} + E_{iso}$	E_{bind}	E_{diff}
Pair	-1175.47	-1162.30	-13.17	-1179.37	-1163.50	-15.87	2.69
Graphitic	-295.99	-280.66	-8.08	-294.20	-281.87	-12.33	4.25
5-9 MV	-649.07	-642.43	-6.65	-650.48	-643.63	-6.85	0.20
s-MV	-645.68	-631.19	-14.49	-650.015	-632.39	-17.62	3.13
5-8-5 DV	-636.31	-622.88	-13.44	-639.87	-624.08	-15.79	2.35
555-777 DV	-857.16	-853.34	-3.82	-862.22	-854.54	-7.68	3.86
TV	-1144.03	-1137.76	-6.27	-1145.59	-1138.97	-6.62	0.35

Supplementary Table 2: Total energy of each configuration in eV when the heteroatom is in the lattice (E_{in}), when the heteroatom is out of the lattice plus the isolated energy ($E_{out} + E_{iso}$), its difference (E_{bind}) and the difference between the binding energies $E_{diff} = E_{bind,O} - E_{bind,N}$

.

STEM image sequences

Video1 is a sequence of STEM images that shows the reduction of the oxygen pair configuration (cf. main text).

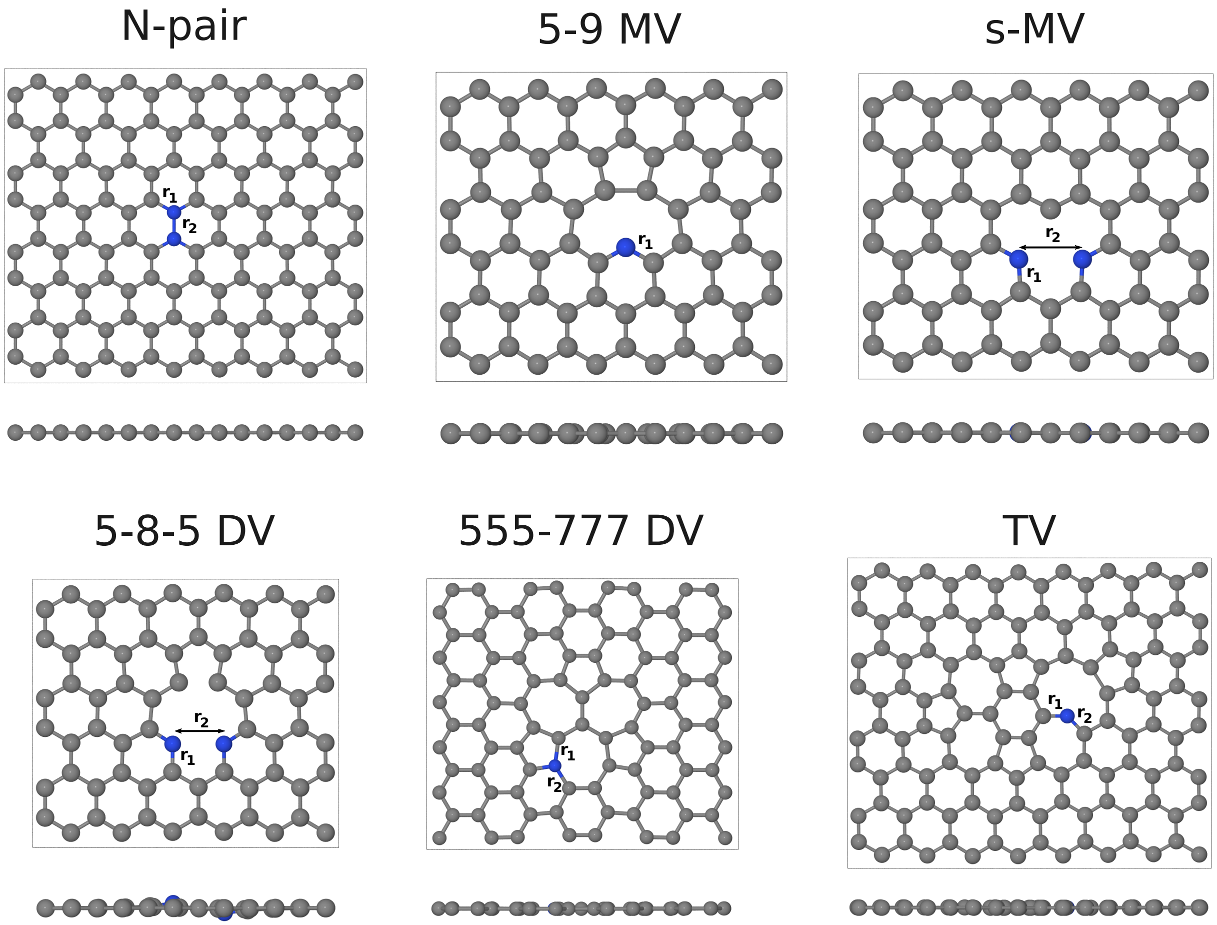

Supplementary Figure 1: Relaxed models for each impurity configuration with nitrogen instead of oxygen.

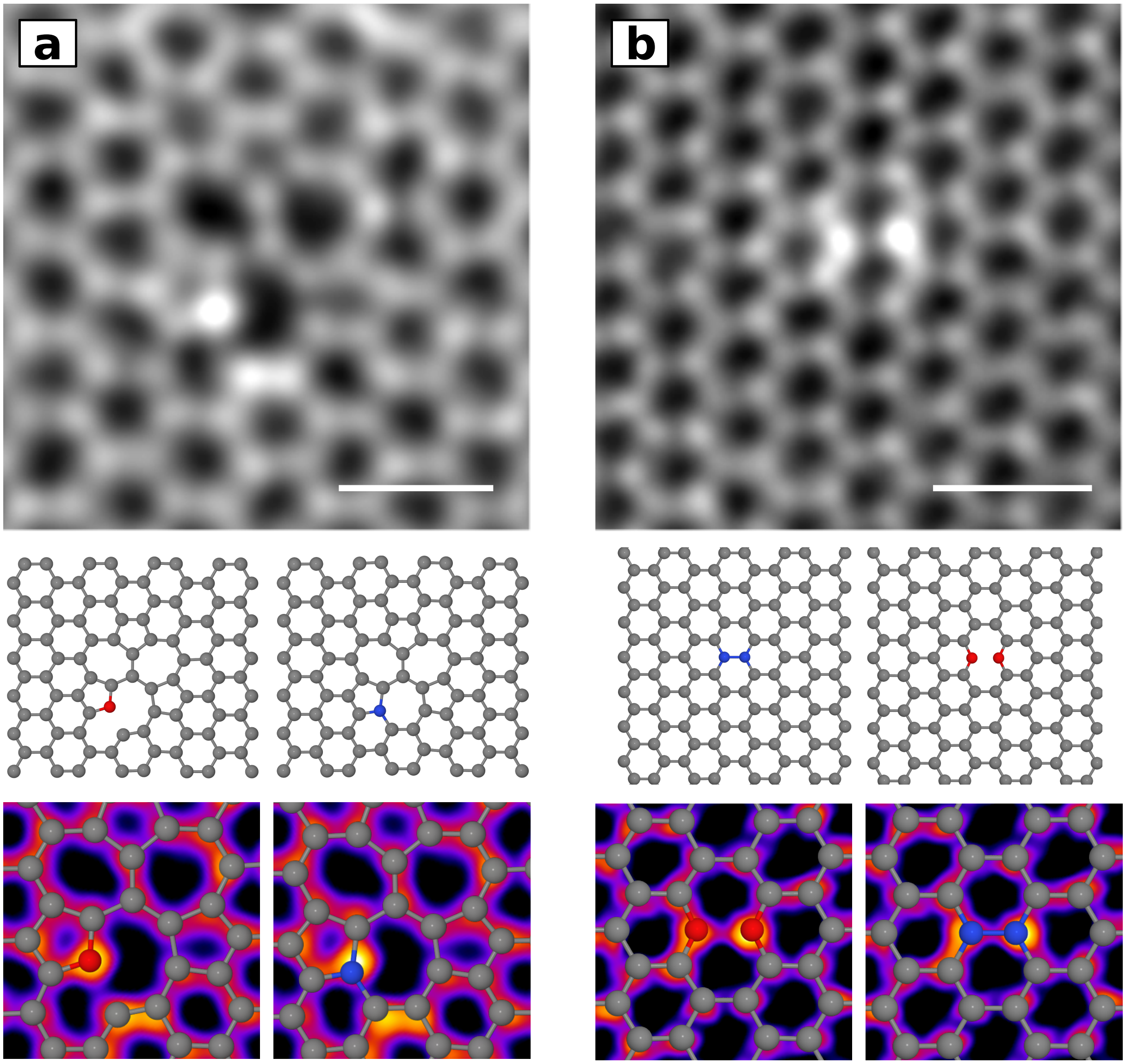

Supplementary Figure 2: Filtered STEM images of (a) the 555-777 DV and (b) the pair configuration. The middle row shows the top view of the relaxed model using oxygen or nitrogen as heteroatoms, respectively. The bottom row shows an overlay of the image from the oxygen sample and the model with oxygen or nitrogen. In both cases, the simulated oxygen configuration matches the experimental image.
