# Supplementary material for: Direct imaging of light-element impurities in graphene reveals triple-coordinated oxygen
Source: Nat Commun. 2019 Oct 8;10:4570. doi: 10.1038/s41467-019-12537-3 (PMC6783479; doi:10.1038/s41467-019-12537-3)
Supplement: Supplementary file 3 — Description of Additional Supplementary Files [file 41467_2019_12537_MOESM3_ESM.pdf]

Description of Additional Supplementary Files

File Name: Supplementary Movie 1

Description: **Reduction of oxygen in graphene oxide**

The video shows a sequence of (false color) STEM images, which reveals the electron beam-induced reduction of oxygen in graphene oxide. Initially, an oxygen pair configuration is present, identified by the two brighter atoms. After several frames, the first oxygen atom gets sputtered leaving a monovacancy, which shows the dynamics as discussed in the manuscript. Then, the second oxygen atom gets kicked out leaving a divacancy.
